# On the relationship between competitive flow and FFT analysis of the flow waves in the left internal mammary artery graft in the process of CABG

**DOI:** 10.1186/s12938-016-0260-4

**Published:** 2016-12-28

**Authors:** Boyan Mao, Wenxin Wang, Zhou Zhao, Xi Zhao, Lanlan Li, Huixia Zhang, Youjun Liu

**Affiliations:** 1 College of Life Science and Bio-engineering, Beijing University of Technology, Beijing, China; 2 Peking University People’s Hospital, Beijing, China

**Keywords:** FFT, CABG, Competitive flow

## Abstract

**Background:**

During coronary artery bypass grafting (CABG), the ratio of powers of the fundamental frequency and its first harmonic (F0/H1) in fast Fourier transformation (FFT) analysis of the graft’s flow waves has been used in the field of evaluation of the patency in anastomosis. But there is no report about using the FFT method to evaluate the magnitude of competitive flow. This study is aiming at exploring the relationship between competitive flow and FFT analysis of the flow waves in left internal mammary artery (LIMA) graft, and finding a new method to evaluate the magnitude of competitive flow.

**Methods:**

At first, establishing the CABG multiscale models of different stenosis in left anterior descending artery (LAD) to get different magnitude of competitive flows. Then, calculating the models by ANSYS-CFX and getting the flow waves in LIMA. Finally, analyzing the flow waves by FFT method and comparing the FFT results with the magnitude of competitive flow.

**Results:**

There is no relationship between competitive flow and F0/H1. As for F0/H2 and F0/H3, they both increase with the reduction of the stenosis in LAD. But the increase of F0/H3 is not obviously enough and it can’t identify the significant competitive flow clearly, so it can’t be used as the evaluation index. It is found that F0/H2 increases obviously with the increase of the competitive flow and can identify the significant competitive flow.

**Conclusion:**

The FFT method can be used in the evaluation of competitive flow and the F0/H2 is the ideal index. High F0/H2 refers to the significant competitive flow. This method can be used during CABG to avoid the risk of competitive flow.

## Background

The flow waves in graft after CABG can reflect whether the surgery is successful. The average flow (Qm), pulsatility index (PI), diastolic fraction (DF), the ratio of the diastolic peak flow and systolic peak flow (D/S) and the percentage of backflow (%Insuf) derived from the flow waves are the important indexes in evaluating the efficiency of the surgery [1–4]. Currently, there have been studies finding that the F0/H1 derived from FFT analysis of flow waves in grafts can evaluate the quality and graft patency, and it is more accurate and reliable than the above indexes [5–8]. Because of the good performance of Fast Fourier transformation (FFT) method, the FFT ratio has been used to differentiate patent grafts [3] and it is put forward as one of the modern diagnostic tools in intraoperative graft patency verification [9].

Competitive flow is the main risk factor influencing the artery graft after CABG [10, 11]. It can decline the flow and generate backflow in the graft, and cause the graft failure due to the string phenomenon [12–15]. So, how to evaluate the competitive flow is a serious problem which researchers and surgeons most concerned. The most effective method evaluating the competitive flow is to clamp the stenosis coronary artery and observe the change of the graft’s flow wave. The significant competitive flow is identified if the flow increases obviously [16]. But, clamping the patient’s coronary artery during the surgery is a dangerous action, it might cause the plaque fall off and block downstream vessel, and it will injure the myocardium. Therefore, evaluating the competitive flow by clamping the stenosis coronary artery is forbidden in clinic. It is necessary to find a method to evaluate the competitive flow without injuring the myocardium. In view of the outstanding performance of the FFT method in evaluating the patency of the anastomosis, this study is going to explore the possibility of using the FFT method to evaluate the magnitude of competitive flow.

Because of the danger of the clinical trials and the difficulty of controlling the graft flow only affected by the magnitude of competitive flow, this study chooses the method of constructing CABG multiscale models of different stenosis. Multiscale models not only guarantee the real geometry of the coronary artery, but also provide precise boundary condition [17, 18]. It has a high accuracy of simulating the physiological conditions, and it has been used in the calculation of various vessels’ hemodynamics. Multiscale models can change the stenosis ratio while other conditions keep constant, and obtain flow waves from models of different stenosis. As there is significant correlation between stenosis and competitive flow, these models can reflect different magnitude of competitive flow.

This study is aiming at exploring the relationship between competitive flow and FFT analysis of the flow waves in LIMA graft, and finding a new way to evaluate the magnitude of the competitive flow by FFT method.

## Methods

### The CABG multiscale model

The multiscale model is constructed by different dimensional models. In this study, the 0D/3D coupling method is used to perform a numerical simulation by coupling the lumped parameter model (0D model) and 3D model [19].

### The reconstruction of 3D model

The patient data is from a male whose cardiac output is 4.6 l/min measured by Doppler ultrasound. The CT data for reconstruction has 460 slices, each of this slice is 512 * 512 pixels, and the distance between each adjacent slice is 1 mm. The patients signed informed consent forms. The threshold segmentation and manual segmentation has been used for reconstruction in Mimics software, and the Gaussian filtering is adopted for smoothing the model surface. Using the Freeform software (software of the 3D modeling system), the model is made different stenosis (100, 90, 75, 60, 50 and 40%) in LAD and completes the LIMA-LAD bypass. The diameter of LIMA is set up as 3 mm. Finally, all of the six models with different stenosis need to be meshed to generate the computational models. The ANSYS-CFX software is used to generate hexahedral mesh in models, and the mesh is refined in the areas of interest to make the simulation results more precise. The node and element numbers for the models are listed in Table 1.

**Table 1:** The nodes and elements numbers of the 3D models

Model (%)	Nodes	Elements
100	1,109,466	1,450,211
90	1,103,681	1,414,879
75	1,081,759	1,366,195
60	1,039,353	1,334,604
50	1,025,067	1,313,895
40	1,013,483	1,304,175

In the 3D simulation, it is assumed that the vessel wall is rigid and the blood is incompressible viscous Newtonian fluid. The density of the blood is 1050 kg/m^3^, and the dynamic viscosity is 0.0035 Pa s.

### The 0D/3D coupling model

**Fig. 1:**
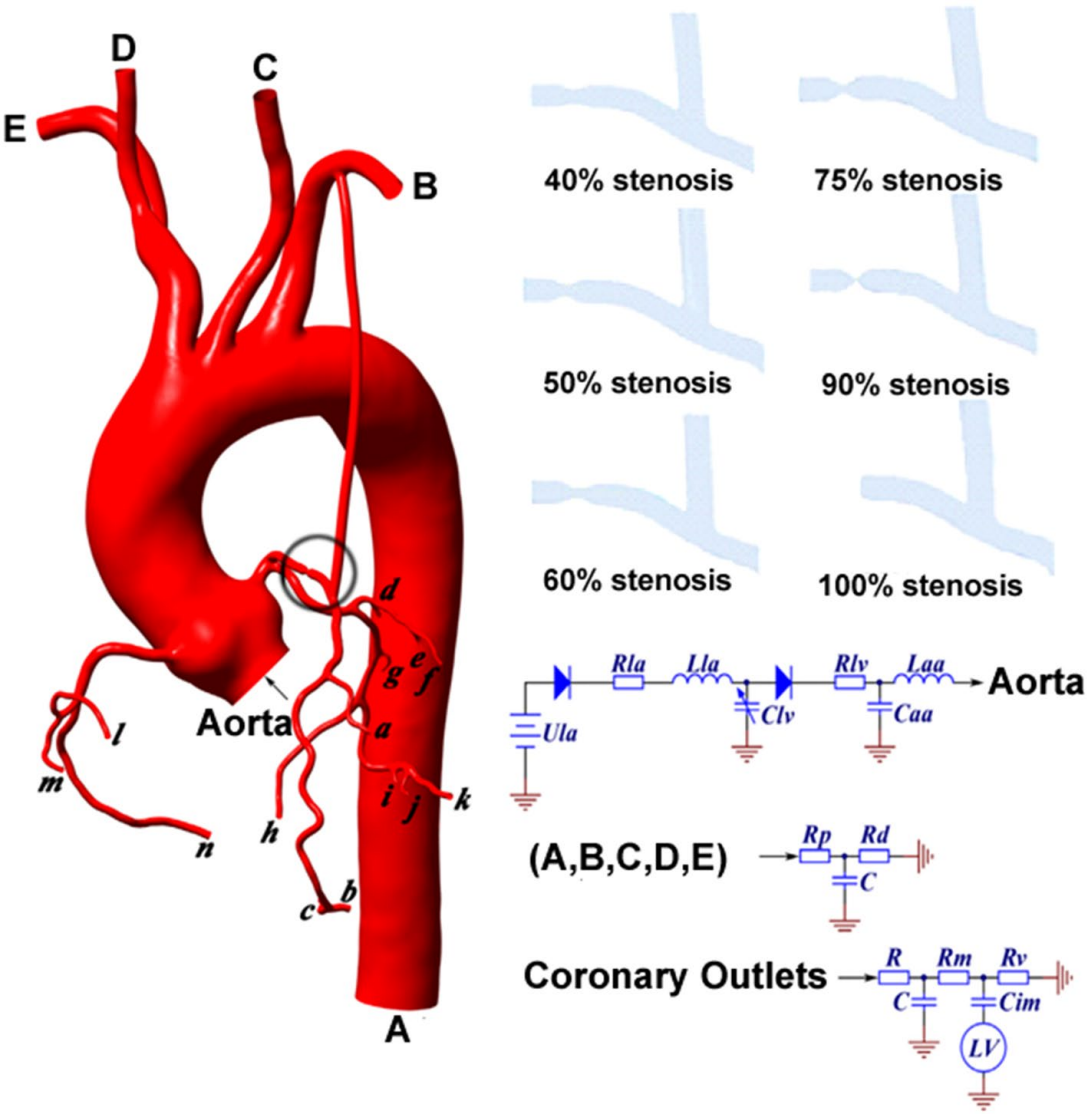
The CABG multiscale model. We construct six models which are similar except for stenosis ratio

In this study, the 0D model which act as the boundary condition for 3D model is described by Taylor [18]. The complete 0D/3D coupling model (multiscale model) is shown as Fig. 1.

The boundary condition of the 3D model are supplied by the 0D calculation and the forcing terms of the 0D model are calculated by the 3D simulation. All of the six 3D models shares the same 0D models, so the difference in the simulation can be caused only by the difference of the stenosis.

As shown in Fig. 1, the inlet of the 3D model (Aorta) is coupled with a 0D model of the heart block. The systemic outlets (A, B, C, D, E) are coupled with a 0D model of the systemic block. And the coronary outlets (a-n) are coupled with a 0D model of the coronary block. In each block, the resistance (R) is used to simulate the flow resistance, the capacitance (C) is used to simulate the compliance of the vessel and the inductance (L) is used to simulate the inertia of the blood flow. In the heart block, the diode is used to simulate the valve in the heart while the variable capacitors Clv is used to simulate the left ventricles. In the coronary block, the pressure of left ventricle is added at the cathode of capacitance Cim which accounts for the intramyocardial pump [18].

In the heart block, the pressure–volume relationship of the ventricle can be described as the following functions.

1 $${\text{Clv}} = \frac{1}{E(t)}$$

2 $${\text{E}}\left( {\text{t}} \right) = \frac{P(t)}{{V\left( t \right) - V_{0} }}$$

where E(t) is the time-varying elastance (mmHg/ml), V(t) and P(t) are the ventricle volume (ml) and pressure (mmHg) respectively, and *V*_0_ (ml) is the reference volume. Mathematically, the function can be approximated as:

3 $${\text{E}}\left( {\text{t}} \right) = (E_{max} - E_{min} )\cdot E_{n} (t_{n} ) + E_{min}$$

where *E*_*n*_(*t*_*n*_) is the normalized time-varying elastance [20].

4 $$E_{n} \left( {t_{n} } \right) = 1.55\left[ {\frac{{\left( {\frac{{t_{n} }}{0.7}} \right)^{1.9} }}{{1 + \left( {\frac{{t_{n} }}{0.7}} \right)^{1.9} }}} \right]\left[ {\frac{1}{{1 + \left( {\frac{{t_{n} }}{1.17}} \right)^{21.9} }}} \right]$$

where $t_{n} = \frac{t}{{T_{max} }}$, *T*_*max*_ = 0.2 + 0.15*t*_*c*_, and *t*_*c*_ is one cardiac cycle interval (s). In this paper, it is set that *E*_*max*_ = 2.0, *E*_*min*_ = 0.002458, *t*_*c*_ = 0.8 s.

The values of parameters in the 0D model are based on data from the research of coronary artery model by Kim [21]. In Kim’s process, it adjusts the parameters of the heart block to make the cardiac output and the aortic pressure calculated by the model fit the clinical measuring values. The total flow of coronary artery is 4.0% of the cardiac output. And that the flow distribution in each coronary branch is consistent with its diameter. Adjusting the parameters of every coronary branch to fit above flow rules. In our models, we set Kim’s parameters’ data as initial values, and cardiac output and aortic pressure are set as objective functions. By using the genetic algorithm, these values are adjusted to make sure that the cardiac output, the systolic and diastolic pressures matches the clinical data, All of the values of parameters are shown in Table 2. Table 3 demonstrates the comparison of the cardiac output, the systolic and diastolic pressures between model predictions and clinical data. It is known that the model data and clinical data have a satisfactory similarity which means the values of parameters are appropriate.

**Table 2:** The values of parameters in 0D model

Heart block	Rla	Rlv	Lla	Laa	Caa
	0.00375	0.0075	0.00285	0.0037	0.95
Systemic block	Rp	Rd	C		
A	0.17	1.517	2.2653		
B	1.311	9.565	0.159		
C	1.312	9.201	0.1588		
D	1.059	9.725	0.09239		
E	1.117	9.325	0.09233		
Coronary block	R	Rm	Rv	C	Cim
a	14.258	129.513	45.254	0.00124	0.01269
b	20.254	135.505	44.002	0.00137	0.00909
c	20.254	135.505	44.002	0.00137	0.00909
d	46.753	110.004	18.751	0.00101	0.0354
e	46.753	110.004	18.751	0.00101	0.0354
f	46.753	110.004	18.751	0.00101	0.0354
g	127.51	207.767	65.255	0.00049	0.00956
h	46.009	156.766	48.505	0.00064	0.01941
i	150.013	264.771	83.256	0.0001	0.00927
j	120.613	211.271	66.456	0.00021	0.01427
k	150.013	264.771	83.256	0.0001	0.00927
l	13.601	155.516	44.505	0.00109	0.0092
m	18.601	145.516	44.505	0.00109	0.0092
n	16.516	136.276	42.008	0.0011	0.01365

**Table 3:** The comparison between model predictions and clinical data

	Model predictions	Clinical data
Systolic pressure (mmHg)	147.69	147
Diastolic pressure (mmHg)	103.48	103
Cardiac output (l/min)	4.58	4.6

Now, the complete CABG multiscale model has been constructed and can be calculated by ANSYS-CFX. The flow waves are extracted from LIMA of the six models for the following analysis.

### FFT analysis to flow waves

Fast Fourier transformation method is a classic signal processing method. It is valuable for clinic using this method to analyze the graft flow waves, and currently, this method has been used in evaluating the patency of anastomosis.

**Fig. 2:**
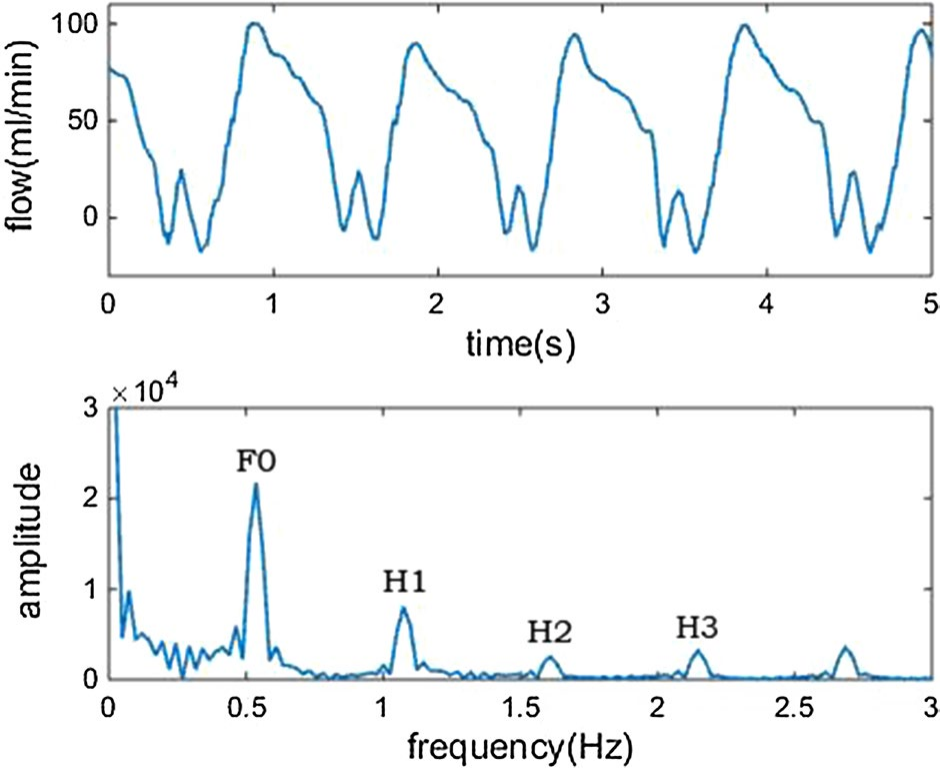
The *first* picture is the LIMA flow waves from a patient who underwent LIMA-LAD bypass grafting; the *second* one is the spectrogram of the FFT analysis

The powers of different frequencies will be obtained through FFT analysis, just as Fig. 2.

In the spectrogram, the first peak is the powers of fundamental frequency (F0), the second peak is the first harmonic (H1). And then, the following are the second harmonic (H2) and the third harmonic (H3), etc.

The FFT analysis of the LIMA flow waves from different stenosis models have been done to explore the possibility of using this method to evaluate the competitive flow.

To prevent the spectrum leakage, the periodic extension is adopted and we take 10 cycles for calculation. In this FFT analysis, the sampling frequency is set up as 50 Hz and the number of sampling points is 2048.

## Results

### The relation of stenosis ratio and competitive flow

**Fig. 3:**
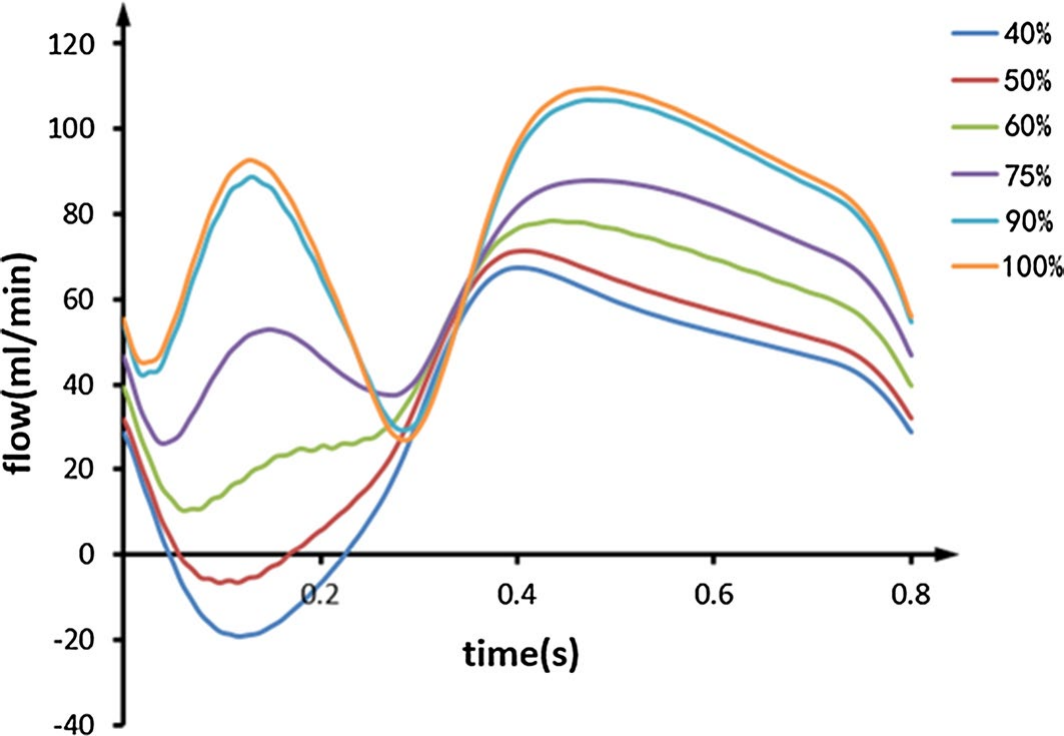
The LIMA flow waves of different stenosis (take one cycle)

In our CABG models, the flow in stenosis LAD and LIMA are gotten. The LIMA flow waves of different stenosis are shown in Fig. 3.

From the picture, it is known that the graft flow decreases with the reduction of the stenosis ratio. Furthermore, the backflow appears when the stenosis ratio is 50%. The presence of backflow means the increase of the oscillatory shear index (OSI) which is a dangerous hemodynamic index for the efficiency of the graft.

The ratio of the flow of the stenosis LAD and LIMA is used to evaluate the magnitude of competitive flow. The high ratio means high magnitude of competitive flow. The flow of stenosis LAD and LIMA, and the ratio of the flow of LAD and LIMA are recorded in Table 4.

**Table 4:** The LIMA flow, the stenosis LAD flow and LAD/LIMA ratio

Model (%)	LIMA flow (ml/min)	Stenosis LAD flow (ml/min)	LAD/LIMA
100	79.2	0	0
90	77.3	2.44	0.03
75	62.3	17.4	0.28
60	50.1	29.8	0.59
50	38.5	41.7	1.08
40	32.5	47.9	1.47

As is known from the Table 4, with the reduction of the stenosis, the LAD/LIMA ratio is gradually increasing. This means the magnitude of competitive flow increases. The ratio has a significant skip when the stenosis is 50%. In this stenosis, the flow in stenosis LAD exceeds the flow in LIMA.

### The FFT analysis of different stenosis

**Fig. 4:**
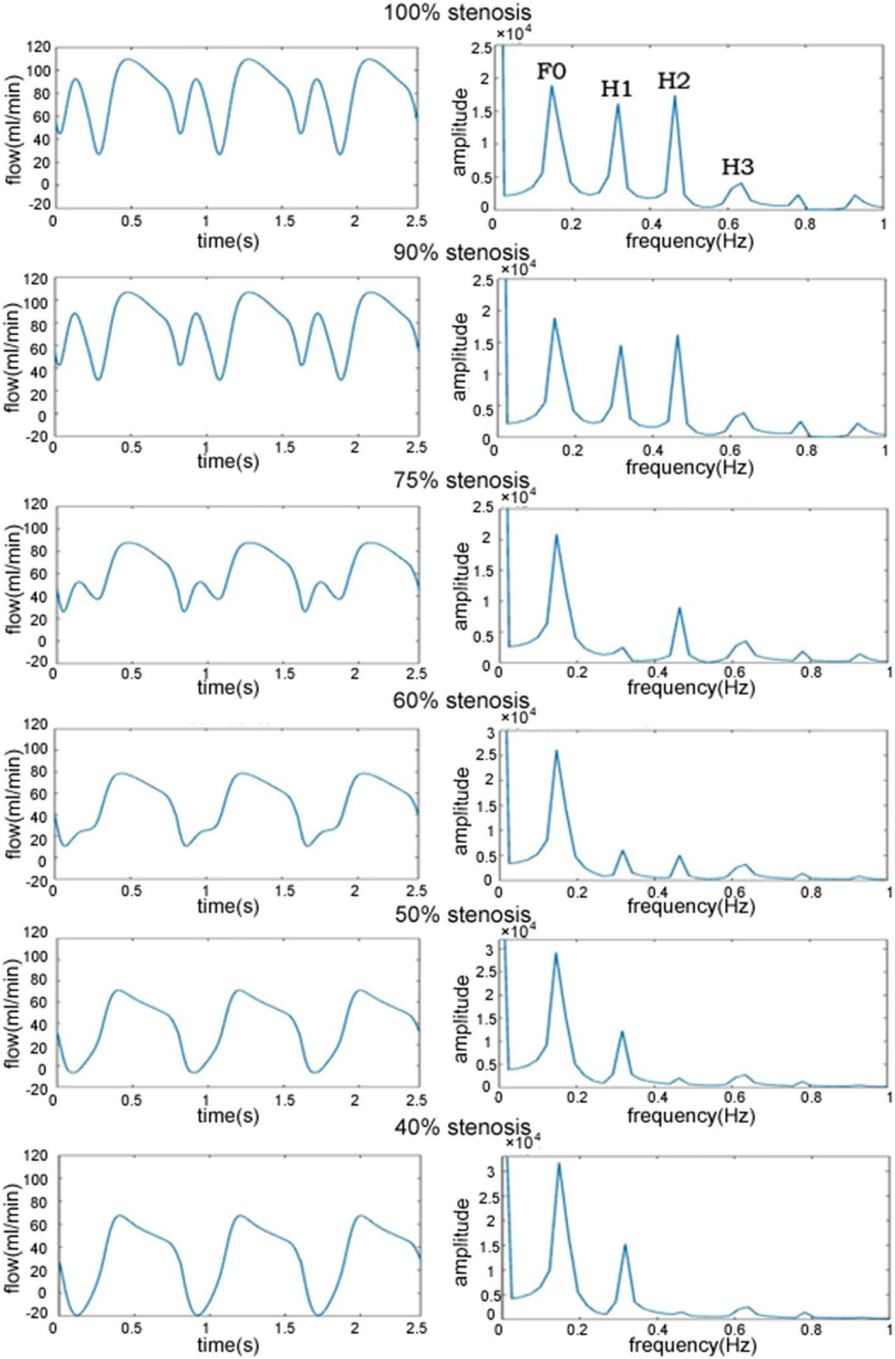
The *left* is the LIMA flow waves of different stenosis, the *right* is the corresponding FFT results

The FFT results of different stenosis have shown in Fig. 4.

Recording F0, H1, H2, H3 of different stenosis, and then calculate F0/H1, F0/H2, F0/H3. These values are shown in Table 5.

**Table 5:** Recording of FFT results

(%)	F0	H1	H2	H3	F0/H1	F0/H2	F0/H3
100	18,920	16,130	17,380	4054	1.17	1.09	4.67
90	18,810	14,510	16,160	3827	1.3	1.16	4.92
75	20,850	2464	9019	3490	8.46	2.31	5.97
60	26,120	6043	5031	3087	4.32	5.19	8.2
50	29,220	12,270	1980	2704	2.38	14.76	10.81
40	31,730	15,290	1449	2502	2.08	21.9	12.68

**Fig. 5:**
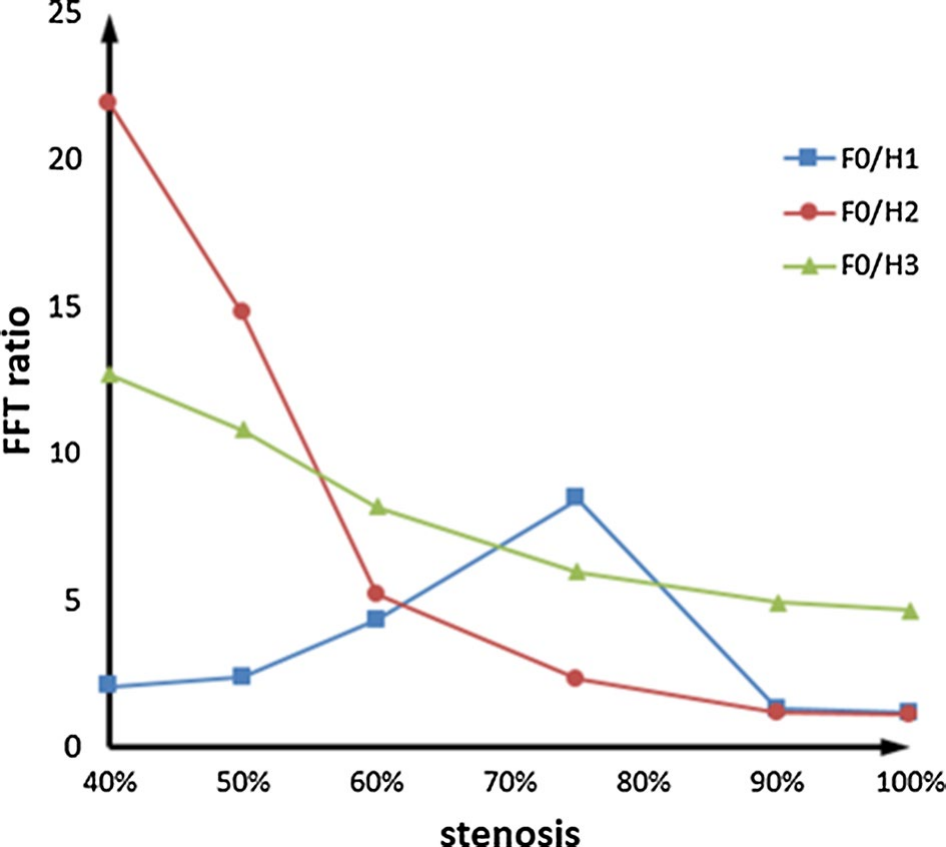
The stenosis-varying* line chart* of FFT ratio

According to Fig. 4 and Table 5, it is known that F0/H1 increases when the stenosis is more than 75%, and then the ratio decreases. F0/H2 and F0/H3 both increase with the reduction of the stenosis. However, the increasing rate of F0/H2 is faster, and there is an obvious skip in 50%. Drawing the stenosis-varying line chart of F0/H1, F0/H2 and F0/H3, shown as Fig. 5.

From the Fig. 3 and Table 4, we know that the magnitude of competitive flow increases with the reduction of the stenosis. Moreover, there is a significant skip of competitive flow accompany with backflow in 50%. In the light of Fig. 5, it is known that there is no relationship between F0/H1 and the magnitude of competitive flow, while F0/H2 and F0/H3 both increase with the increase of the competitive flow. But the curve of F0/H3 is too gentle to distinguish the different magnitude of competitive flow. The F0/H2 curve has an obvious difference among different magnitude of competitive flow, and has a significant skip in 50% compared to 60% which is consistent with the actual situation. Therefore, the index of F0/H2 can reflect the magnitude of competitive flow commendably.

## Discussion

In this paper, we use the 0D/3D coupling model method to construct a CABG model of different stenosis. It can ensure that LIMA flow waves only influenced by stenosis ratio. By comparing stenosis LAD flow and LIMA flow, it can be found that the magnitude of competitive flow increases with the reduction of stenosis ratio. And the competitive flow has a significant increase accompany with the backflow in 50% stenosis.

By doing FFT analysis to LIMA flow waves of different stenosis, we can get F0/H1, F0/H2 and F0/H3. Finding that F0/H1 and F0/H3 have defects in evaluating the competitive flow, while F0/H2 has an outstanding performance. The F0/H2 increases obviously with the increase of the competitive flow and can distinguish the significant competitive flow in 50% stenosis. So the F0/H2 can be used as an effective index in evaluating the competitive flow.

Therefore, to evaluate the competitive flow, the FFT analysis should be taken and F0/H2 is calculated after obtaining LIMA flow waves in clinic. High F0/H2 suggests significant competitive flow. This method avoids defects existed in the method which needs to clamp the stenosis LAD. It can evaluate competitive flow effectively while there is no damage to the heart and instruct surgeons to take measures to avoid the harm of competitive flow.

However, there is no reports published about the physiological meaning of FFT ratio. In our study, it is found that F0/H1 might be related to the ratio of systolic peak flow and diastolic peak flow, while F0/H2 and F0/H3 are lack of research. For future work, it is necessary to study the physiological meaning of FFT ratio and this will help researchers recognize and improve the method.

## Conclusion

The FFT analysis could be used to evaluate the magnitude of competitive flow and F0/H2 is the index which is more effective than F0/H1 and F0/H3. High F0/H2 suggests significant competitive flow, while low F0/H2 suggests light competitive flow.

## Abbreviations

CABG: coronary artery bypass graft
FFT: fast Fourier transformation
LIMA: left internal mammary artery
LAD: left anterior descending artery
F0/H1: the ratio of powers of the fundamental frequency and its first harmonic
F0/H2: the ratio of powers of the fundamental frequency and its second harmonic
F0/H3: the ratio of powers of the fundamental frequency and its third harmonic

## References

[1] Tokuda Y, Song MH, Ueda Y (2007). Predicting early coronary artery bypass graft failure by intraoperative transit time flow measurement. Ann Thorac Surg.

[2] Tokuda Y, Song MH, Oshima H (2008). Predicting midterm coronary artery bypass graft failure by intraoperative transit time flow measurement. Ann Thorac Surg.

[3] Kim KB, Kang CH, Lim C (2005). Prediction of graft flow impairment by intraoperative transit time flow measurement in off-pump coronary artery bypass using arterial grafts. Ann Thorac Surg.

[4] Di Giammarco G, Pano M, Cirmeni S (2006). Predictive value of intraoperative transit-time flow measurement for short-term graft patency in coronary surgery. J Thorac Cardiovasc Surg.

[5] Takami Y, Ina H (2001). Relation of intraoperative flow measurement with postoperative quantitative angiographic assessment of coronary artery bypass grafting. Ann Thorac Surg.

[6] Une D, Chikazawa G, Karkhanis R (2011). Can Fast Fourier Transformation (FFT) analysis of graft flow predict 1 year graft failure after CABG?. Can J Cardiol.

[7] Hatada A, Yoshimasu T, Kaneko M (2007). Relation of waveform of transit-time flow measurement and graft patency in coronary artery bypass grafting. J Thorac Cardiovasc Surg.

[8] Takami Y, Ina H (2001). A simple method to determine anastomotic quality of coronary artery bypass grafting in the operating room. Vascular.

[9] Fabio Bartolozzi MD, Michele Pilato MD (2009). Intraoperative graft patency verification in coronary surgery: modern diagnostic tools. Intraoperative Flow Meas Coron Artery Surg.

[10] Sabik JF, Lytle BW, Blackstone EH (2003). Does competitive flow reduce internal thoracic artery graft patency?. Ann Thorac Surg.

[11] Pagni S, Storey J, Ballen J (1997). Factors affecting internal mammary artery graft survival: how is competitive flow from a patent native coronary vessel a risk factor?. J Surg Res.

[12] Villareal RP, Mathur VS (2000). The string phenomenon: an important cause of internal mammary artery graft failure. Tex Heart Inst J.

[13] Beijk MA, Harskamp RE. Treatment of coronary artery bypass graft failure. In: Aronow WS (ed) Artery bypass. InTech; 2013. p. 193–237.

[14] Barner HB (1974). Double internal mammary-coronary artery bypass. Arch Surg.

[15] Seki T, Kitamura S, Kawachi K (1992). A quantitative study of postoperative luminal narrowing of the internal thoracic artery graft in coronary artery bypass surgery. J Thorac Cardiovasc Surg.

[16] Bolotin G, Kypson AP, Nifong LW (2003). A technique for evaluating competitive flow for intraoperative decision making in coronary artery surgery. Ann Thorac Surg.

[17] Sankaran S, Moghadam ME, Kahn AM (2012). Patient-specific multiscale modeling of blood flow for coronary artery bypass graft surgery. Ann Biomed Eng.

[18] Taylor CA, Fonte TA, Min JK (2013). Computational fluid dynamics applied to cardiac computed tomography for noninvasive quantification of fractional flow reserve: scientific basis. J Am Coll Cardiol.

[19] Zhao X, Liu Y, Li L (2016). Hemodynamics of the string phenomenon in the internal thoracic artery grafted to the left anterior descending artery with moderate stenosis. J Biomech.

[20] Stergiopulos N, Meister JJ, Westerhof N (1996). Determinants of stroke volume and systolic and diastolic aortic pressure. Am J Physiol Heart Circ Physiol.

[21] Kim HJ, Vignon-Clementel IE, Coogan JS (2010). Patient-specific modeling of blood flow and pressure in human coronary arteries. Ann Biomed Eng.

